# How do Android developers improve non-functional properties of software?

**DOI:** 10.1007/s10664-022-10137-2

**Published:** 2022-05-30

**Authors:** James Callan, Oliver Krauss, Justyna Petke, Federica Sarro

**Affiliations:** 1 University College London, London, UK; 2 University of Applied Sciences Upper Austria, Wels, Austria

**Keywords:** Non-Functional property optimisation, Android optimisation, Mining android, Execution time, Bandwidth, Framerate, Memory

## Abstract

Nowadays there is an increased pressure on mobile app developers to take non-functional properties into account. An app that is too slow or uses much bandwidth will decrease user satisfaction, and thus can lead to users simply abandoning the app. Although automated software improvement techniques exist for traditional software, these are not as prevalent in the mobile domain. Moreover, it is yet unknown if the same software changes would be as effective. With that in mind, we mined overall 100 Android repositories to find out how developers improve execution time, memory consumption, bandwidth usage and frame rate of mobile apps. We categorised non-functional property (NFP) improving commits related to performance to see how existing automated software improvement techniques can be improved. Our results show that although NFP improving commits related to performance are rare, such improvements appear throughout the development lifecycle. We found altogether 560 NFP commits out of a total of 74,408 commits analysed. Memory consumption is sacrificed most often when improving execution time or bandwidth usage, although similar types of changes can improve multiple non-functional properties at once. Code deletion is the most frequently utilised strategy except for frame rate, where increase in concurrency is the dominant strategy. We find that automated software improvement techniques for mobile domain can benefit from addition of SQL query improvement, caching and asset manipulation. Moreover, we provide a classifier which can drastically reduce manual effort to analyse NFP improving commits.

## Introduction

Several studies have shown that non-functional performance characteristics have a strong impact on user satisfaction (Inukollu et al. 2014; Lim et al. 2014; Khalid et al. 2014; Liu et al. 2014; Ferrucci et al. 2015; Kim et al. 2015; Martins et al.^2018^; Gao et al. 2018, 2020).

Performance issues are especially important in the mobile, resource constrained domain. Banerjee and Roychoudhury (2017) analysed 170,000 user reviews of mobile applications, and classified reasons for user downvotes. Three out of five identified categories related to non-functional properties. Khalid et al. (2014)’s study of iOS applications also showed that unresponsive, resource heavy applications, and those with network related issued were among the top most frequent sources of complaints. Issues related to non-functional software properties lead not only to downvotes, but also large number of uninstallations (Banerjee and Roychoudhury 2017) rendering them a priority in the mobile software development process.

Most of the aforementioned work provides analysis on what the issues are, rather than *how* these can be solved. Only recently Hort et al. (2021) carried out a comprehensive survey of existing research on performance optimisation for mobile applications. These included offloading, antipattern detection, refactoring, prefetching, choice of a different programming language, reordering of I/O calls and changes to hardware components (Hort et al. 2021). Their survey shows that tool support for automated improvement of non-functional properties has been scarce, mainly targeting energy consumption, and if available, targeting one property of choice, sometimes having adverse effects on the others (Hort et al. 2021).

Among automated approaches for improvement of non-functional properties for traditional software, search-based approaches have gained popularity in the last few years. For example, Wu et al. (2015) automatically applied changes directly to source code to produce a Pareto front of program variants that improve memory consumption and runtime efficiency. Basios et al. (2018) and Burles et al. (2015) both modified the data structures used by software to improve non-functional properties by finding less resource-demanding combinations. These and other techniques have been successful at optimising traditional software, though these are yet to be applied more widely in the mobile domain. The advantage of using search-based tools, is that they are well-suited for multi-objective optimisation, something that is missing in the mobile application improvement field (Hort et al. 2021).

We pose that software repositories offer researchers a wealth of information about the behaviour and techniques used by actual developers. These can be used to find patterns that can be mimicked by search-based software engineering approaches for optimisation of non-functional software properties (Harman and Jones 2001). Although several previous studies focus on performance bugs in traditional software, such as the study by Jin et al. (2012a), only a few studies on mining performance improving commits in the Android domain exist (e.g., Das et al. 2016; Moura et al. 2015). Moreover, those do not provide fine-grained enough information to guide developers of search-based software development tooling. Previous studies were also concerned with finding general patterns across as many projects as possible, thus employed a sampling strategy that would alleviate the expensive manual analysis cost. This leads to under-approximation of the true number of non-functional property improving changes.

To fill this gap we mined the most popular Android repositories, using single-keyword search and analyse all returned results, to find patterns that could be utilised in search-based automated software improvement tooling. We focus on four non-functional properties in particular: execution time, memory consumption, bandwidth usage, and frame rate. We chose these as they are most related to mobile app performance, key issue for users, as previous studies show Banerjee and Roychoudhury (2016) and Hort et al. (2021).

First, we mined the repositories of the 20 most popularly downloaded mobile applications, according to Fossdroid, and manually examine the resultant 3,132 commits,finding 229 were actually NFP improving ones.^1^ Although this process should give us a good overview of non-functional property improving strategies for performance, it only allows for analysis of a relatively small number of repositories. However, the detailed analysis provides us with a corpus of data on which we can train a classifier that could help gather and analyse more data. Therefore, we devised such a classifier and analysed a further randomly selected set of 80 repositories, manually analysing 495 commits found, which added 331 non-functional property improving commits to our dataset. We categorised all the commits found, to help us identify emerging patterns. We also report on whether current automated improvement tools already allow for such transformations to be found, and if not, if such tools could be extended to provide new, useful software transformations. Finally, we examined features of the repositories we analysed. This is to provide recommendations for software developers, for what types of mobile applications non-functional improvements are likely to be found.

Our results show that non-functional property improvements to app performance are rare: from 74,408 commits mined across 100 repositories, only 560 were deemed to improve execution time, memory consumption, frame rate or bandwidth (229 identified by manual search and 331 by using a classifier). However, we can still draw interesting conclusions about their nature. In particular:

- In 10.7% of cases, developers were willing to sacrifice one non-functional property over another, while in 6.5% of cases developers were able to improve upon multiple properties at once. This shows the need for tooling that can handle multi-objective optimisation.
- The strongest indicators for the number of non-functional property improving commits in a repository was the total number of commits, number of contributors and number of stars.
- Current search-based improvement tooling mimics 5 out of 23 non-functional improvement strategies found.
- Future automated techniques for improvement of non-functional properties could be enhanced by incorporating automated caching, SQL query, and image transformations. We propose detailed transformation patterns to aid researchers and developers in the design and adoption of such strategies.

Overall our results provide recommendations for software engineers, aiming to provide better tooling for automated software improvement; and for researchers, providing patterns of how developers improve mobile applications’ non-functional properties related to mobile app performance, as well as a classifier that can help with future mining studies in this domain.

All our data and scripts are freely available to allow for reproduction (github.com/SOLAR-group/NonFunctionalAndroidCommits), replication and extension of our work.

The rest of this paper is organised as follows: Section 2 describes our methodology; Section 3 presents our results; in Section 4 we discuss implications of our study in software engineering research and practice; Section 6 presents threats to validity; while Section 5 presents related work; Section 7 concludes the paper. Appendix Appendix contains additional materials.

## Methodology

In order to answer *how Android developers improve the performance-related non-functional properties of software (performance NFPs)*, and how we can use this knowledge to potentially devise new software transformations for tools for automated software improvement, we mine open-source Android projects for commits that improve four non-functional software properties (NFPs): *execution time*, *memory consumption*, *bandwidth usage*, and *frame rate*. Along with energy efficiency, previous research shows these are often found in user reviews (Banerjee and Roychoudhury 2017; Khalid et al. 2014), yet have not been extensively tackled in the literature (Hort et al. 2021).^2^

We aim to answer the following research questions:

**RQ1**: *With what prevalence do developers improve performance NFPs of Android apps?*
  NFPs of mobile applications impact user satisfaction, however it is not clear to what extent Android developers change their code to improve performance NFPs. The aim of this question is twofold: understanding if there exist NFP commits in Android open-source repositories to extract general patterns from, and understanding their characteristics.
**RQ2**: *How and when do Android developers improve app’s performance NFPs?*
  We want to know at which stage in software development do performance NFP improving commits occur, whether these are considered as standalone improvements, and whether these improve multiple NFPs or prioritise one whilst possibly sacrificing another. These should give us an overview of the current Android development practice with respect to performance NFP improvement.
**RQ3**: *What type of code changes do Android developers make to improve app’s performance NFPs?*
  We want to also investigate what sort of changes developers make to source code to improve its performance NFPs. Examining these changes will allow us to compare current search-based improvement techniques to real-world commits and make suggestions for how these techniques can be improved.

To answer these research questions we have manually curated a corpus of 560 non-functional property improving commits, which were collected by analysing a total of 74,408 commits mined from 100 open-source Android repositories. In the following section we explain in detail our collection procedure. We have made this corpus publicly available to allow for replication and extension of our work (github.com/SOLAR-group/NonFunctionalAndroidCommits).

### Overview of Methodology

Below we present the methodology used to create our corpus. It consists of three steps:

**Keyword mining:** In this step we collect a set of performance NFP improving commits by filtering them first based on keywords and then manual analysis.
**Classifier mining:** In this step we expand this set by using a classifier trained on the commit messages gathered in the previous step.
**Categorisation:** In this step we attempt to manually group the commits into categories. These categories allow us to find common patterns used to improve the four non-functional properties of interest: runtime, memory consumption, bandwidth and frame rate.

### Corpus

In the first step, we mined the twenty most popularly downloaded Android applications according to Fossdroid,^3^ and extracted a total of of 28,028 commits. As it would have been infeasible to manually inspect such a large set to identify NFP improving commits, we have adopted a semi-automatic approach that examines every commit message based on keyword search (as detailed in Section 2.3). This lead us to a total of 3,132 commits, which were then manually analysed in order to label them as performance NFP improving commits or not. A final set of 229 NFP improving commits was deemed to improve one of the four non-functional properties of interest. We note that in previous work Moura et al. (2015) opted for two-word key-phrases rather than keywords to massively narrow down the number of commits to manually analyse. Das et al. (2016) only mined commits from the main modules of applications, missing any changes to back-end modules. We opted not to take these actions, and avoid missing possible useful software transformations by mining all commits with generic keywords.

In the second step, we leverage this curated set of NFP improving commits, to train a classifier to be able to automatically identify such commits. This allowed us to automatically analyse a much larger set of commits (46,378), mined from 80 randomly selected F-droid repositories, and filter out irrelevant (i.e., not NFP improving) commits with a precision of 95%, as detailed in Section 2.4. Specifically, we used the classifier to automatically identify 331 additional NFP improving commits by randomly sampling F-droid. We initially found a total of 495 commits, which were then manually validated by two of the authors to make sure they improve any of the four non-functional properties of interest. This manual check led to the identification of 331 performance NFP improving commits.

The final size of our manually curated corpus thus consists of 560 NFP improving commits (229 from the first and 331 from the second step). We then manually categorised these commits by the type of change which was made to improve the NFP, by analysing their commit messages and diffs. This resulted in 23 categories of improvement types being found.

Next, we detail how we mine NFP improving commits by using keyword search (Section 2.3) and the classifier (Section 2.4), as well as how we manually validate the NFP improving commits and categorise them (Section 2.5).

### Step 1: Identifying NFP Improving Commits Based on Keyword Search

We mined 28,028 commits from the twenty most popularly downloaded applications according to Fossdroid (as of 18/03/2020), a website which offers an alternative user interface to the standard F-Droid web page. These applications are diverse in nature (e.g., gaming applications, streaming applications, browsers) and size, having between 13 and 6,157 commits. Details of each application repository can be found in Tables 1 and 5. Whilst the repositories of these applications are hosted on a variety of platforms (GitHub, GitLab, etc.), all repositories use the git version control system. The git log command was used to generate a list of commit messages, which was then parsed and searched for sets of relevant commits, that suggest improvements to the following four non-functional properties:

1. Execution Time: Decreasing the amount of time needed for computation.
2. Memory Consumption: Decreasing the amount of RAM used.
3. Bandwidth Usage: Reduction of the load on the network.
4. Frame Rate: Decreasing frame rendering and display rate.

**Table 1:** Properties of repositories mined based on keyword search

Repository	Type of App	Comm.	Stars	Age (days)	Contrib.	Forks	KLoC
Aeons End	Game	26	5	963	1	5	3.0
AFH Downloader	Network.	69	18	1407	2	7	2.4
Android CUPS Print	Printing	274	142	1802	14	45	4.9
ANNO 1404	Game	13	1	1127	2	2	5.0
Apple Flinger	Game	463	22	972	37	–	11.8
Calculator	Calculator	1142	190	4220	18	286	32.8
Call Recorder	Audio	1590	97	1523	10	–	6.1
DNS66	Network.	341	1400	1304	15	153	7.8
Editor	Text Editor	405	110	1038	14	38	5.3
F-Droid	App Store	6157	1382	3492	99	–	85.1
Firefox	Browser	2592	1500	1249	82	585	309.2
FOSS Browser	Browser	927	427	1292	22	165	15.9
Frozen Bubble	Game	157	71	3829	4	64	38.4
G-Droid	App Store	625	78	557	60	–	17.2
Gadgetbridge	Network.	5163	74	1587	298	40	103.9
Gloomy Dungeons	Game 2	46	73	2006	4	38	91.2
MaterialOS	Themes	139	117	1834	7	37	14.5
Mi Mangu Nu	Books	1827	230	1839	23	60	33.3
Mighty Knight	Game	18	11	1250	2	12	1.0
NewPipe	Video Stream.	6054	8000	1711	439	1200	82.9

In order to identify relevant performance NFP improving commits, each repository was mined by searching every commit message for a series of keywords (or parts of words in some cases, e.g. “effic” to capture all words similar to “efficient”, “inefficient”, etc.) associated with the particular property, by following a three-step process, as described below, and then manually validated.

#### Initial Selection

An initial set of keywords was generated by a combination of our knowledge of relevant terminology (which we have gained by writing NFP improving commits ourselves) and the examination of the language used in commit messages written by others. We then augmented this set with 15 keywords^4^ used in previous work conducting similar analysis (Jin et al. 2012b; Mazuera-Rozo et al. 2020; Das et al. 2016; Linares-Vásquez et al. 2015; Chen et al. 2019b). Any commit containing any of these keywords was selected for manual evaluation. Every selected commit message was manually evaluated to see if it actually suggests that an NFP has been improved or not. This approach aims to highlight as many commits as possible that could improve non-functional properties and therefore result in many false positives being manually evaluated. This helps to reduce the number of false negatives and allows us to detect as many relevant commits as possible.

#### Keyword Expansion

Synonyms for all keywords were searched for using the SEThesaurus (Chen et al. 2019a), a natural language processing (NLP) tool for finding synonyms in an SE context. Terminology found during manual evaluation of commits which suggests improvement but not present in the initial keyword set was added to a new keyword set. Another search took place with the new keywords in the same way as the original search. The keywords used can be found in Table 2.

**Table 2:** Keywords used to search for commit types, from initial selection and **Keyword Expansion** stages. Note that extensions of keywords are also captured during search, e.g., speeding, performance, and other

Property	Keywords
Execution Time	speed, time, perform, slow, fast, optimi, wait, tim, stuck, react,
	suboptimal, utilization, ANR, bottleneck, hot-spot, length, **effic**
Memory	memory, leak, size, cache, buffer, bloat, consumption, OOM **space**, **storage**
Bandwidth	network, bandwidth, size, download, upload, socket latenc, throughput,
Frame Rate	frame, lag, respons, latenc, **hang**

#### Keyword Validation

To validate the keywords we conducted a text analysis by tokenising and lemmatising all words over all commit messages. The resulting 12,230 tokens were grouped according to the commits *relevant (229)*, *irrelevant (3132 - 229)*, and *filtered out (27028 - 3132)*, based on the keywords used. These tokens were then ranked by how often they occur in each group. From these rankings we attempted to identify possible keywords that we may have missed. First we removed all tokens that occur less than 10 times in the commits identified as improving performance NFPs: This resulted in the identification of 76 tokens, which could potentially be used as keywords. Then we further filtered out tokens by focusing only those that occur in the *relevant* group more or as often than those occurring in the *irrelevant* group. This step allowed us to filter out words such as ‘and’, which are common in all commits. Of the 6 remaining tokens, three were already included as keywords (i.e., *memory*, *faster*, and *leak*). The remaining three were *save, reduce* and *low*. These three terms may be considered as additional keywords to identify additional NFP commits, yet their use could increase the already high manual effort needed to inspect the selected commits. In fact, in our study, these three keywords (save, reduce, low) relate to 111 *filtered out* commits. After manual inspection, we found that of these 111 commits only a single one could be identified as relevant; this commit also contains the word ‘mem’ instead of ‘memory’, suggesting that using keyword search may miss those commits that use abbreviations like this or contain misspellings of keywords. However, as most commits contain more than one keyword, the keyword set used herein can capture the majority of those commits too. As only three more relevant keywords were identified out of the 12,230 unique tokens present in the *relevant* commits, and they led to the identification of only one additional relevant commit out of 111, we are confident that the set of keywords used to conduct our study is comprehensive and effective.

Furthermore, the first author of this paper manually analysed the resultant commit set. Some commit messages were found ambiguous as to whether or not they offer any improvement. Developers sometimes write commit messages about what they have done but not why they have done it. Such commits were also independently analysed by another author. If the second author also found the commit to be ambiguous and not explicitly labelled as and improvement, it was discarded. We also discarded those commits which were merges with a single child commit as they were considered duplicates. We refer to the final set of manually curated commits gathered in this step as the “manual set”.

### Step 2: Identifying NFP Improving Commits Based on Automated Classification

While in the previous step we use keyword search to narrow-down the number of commits for manual investigation, in this step we explore the use of an automated classifier, which leverages on the manual set obtained from Step 1.

The classifier we propose has been trained with the classified data from Step 1, i.e., all commits manually excluded after the keyword search are labelled as *irrelevant*, while all commits included are labelled as *relevant*.^5^ In addition, we have included 368 commits manually identified as relevant towards execution time in previous work (Mazuera-Rozo et al. 2020) to the *relevant* commit set. We train the classifier using only the commit messages of the commit.^6^

In order to search for an accurate prediction model, we have investigated a total of 20 classification algorithms exploiting 6 different settings for feature selection. The settings were derived from the featurization of text tokens via TF/IDF, Bag of Words (Yamauchi et al. 2014), and an adapted version of Bag of Words where only words occurring with a discriminative significance in either the irrelevant or relevant groups were used in the feature vector. Next, we present only the best result of these attempts, while more information about the training of the classifier can be found in Appendix A. The best classifier was achieved via stemming as pre-processing step, TF/IDF for featurization using a Decision Tree classifier. We assessed its effectiveness via cross-validation by using 10 hold-out repetitions (80%/20% train/test split), each time using a different seed. The results show a good level of classification with a precision of 73% and recall of 80% in the *relevant* class (see Table 3).

**Table 3:** Decision tree classification of NFP improving commits allows an accurate classification (0.80 recall) with a tolerable level of irrelevant commits mixed in (0.73 precision)

	Precision	Recall	F1-score
Relevant	0.73	0.80	0.76
Irrelevant	0.95	0.92	0.93

In order to show the reduction in manual effort required when using our classifier we run it on two datasets. Table 4 shows a comparison of commits identified via keyword search or via the classifier. For the dataset from Mazuera-Rozo et al. (2020) we applied the keywords from Table 2, after compiling the git logs from the repositories used in the dataset by Mazuera-Rozo et al. (2020). The table shows that keyword search requires a much higher manual effort as the search returns several thousand keywords (3,132 our dataset and 32,308 from Mazuera-Rozo et al.) containing only a few relevant commits (229 and 368). The classifier returns only 669 commits, with 219 from the manual identified ones contained (only 10 missed), and an additional 440 commits that may be relevant, but were filtered by the keyword search.

**Table 4:** Comparing our keyword search to our classification based approach on two datasets. The *368* number of relevant commits for the Mazuera-Rozo et al. dataset was taken from their work https://github.com/amazuerar/perf-bugs-mobile/blob/master/bug-fixing-commits-performance.csv. We note that authors report 380 in their paper, but 11 commits don’t exist anymore

		Our dataset	Mazuera-Rozo et al. (2020)
	Total commits	28,028	420,352
Our Keyword Search	Identified	3,132	32,308
	Relevant	229	*368*
Classifier	Identified	669	3477
	Relevant	219	355
	Missed	10	13
	Additional	440	3109

As the cross-validation confirms the effectiveness of the classifier, we re-train it on the entire available dataset in order to classify performance NFP improving commits on unseen data, thus further validating our classifier in a real usage scenario, and extending our corpus of NFP improving commits with the commits correctly classified as such.

To this end, we randomly selected 80 repositories from F-Droid and used the classifier to automatically classify all 46,378 commits extracted from these repositories.^7^ Details of the repositories are provided in Table 5. The classifier identified 475 commits *relevant* commits. Two of the authors manually analysed these commits, as they did in Step 1, to check whether the commits classified as relevant are actually NFP improving commits, i.e., true positives. They found that only 164 commits were false positives, giving a manually evaluated precision of 66.87% for this classifier, and 331 commits added to our corpus.^8^

**Table 5:** Properties of classifier mined repositories

Name	Commits	Stars	Age (days)	Contrib.	Forks	KLoC
Alwayson	362	75	882	5	13	10.0
Android-inventory-agent	982	41	3584	14	25	12.2
Android-usb-serial-monitor-lite	50	140	3237	2	77	2.2
Anewjkuapp	1332	12	2423	14	6	27.4
Ankieditor	47	21	1226	2	6	41.6
Atmospherelogger	65	14	3354	1	4	3.0
Audioanchor	243	109	662	12	21	8.3
Audiometer	61	20	1493	3	7	1.3
Ausweisapp2	52	275	1311	9	42	137.6
Autoairplanemode	20	15	1398	3	8	1.9
Avare	1790	114	2940	28	116	55.2
Blexplorer	92	46	2080	4	23	1.7
Boogdroid	189	9	1819	5	3	4.0
Botbrew-gui	135	49	3141	1	15	5.10
Changedetection	219	584	938	11	72	14.9
Cmus-android-remote	45	11	2512	1	5	4.4
Controlloid-client	95	62	683	2	8	15.1
Covid19stats	81	139	273	4	39	2.0
Dailypill	251	5	401	2	2	1.10
Dandelion	651	102	1753	16	36	21.2
Droid48	96	59	3747	2	22	19.2
Easytoken	102	44	2364	1	13	4.2
Easywatermark	125	596	153	5	60	7.10
Gears2	63	17	3521	1	11	3.6
Gigaget	128	205	2219	2	52	5.5
Glesquake	14	15	2312	1	6	150.3
Glt-companion	482	9	2106	5	3	11276.9
Gpodroid	69	25	3523	2	3	3.2
Http-shortcuts	1144	354	2067	8	66	59.10
Headingcalculator	38	1.2	2329	1	1	1.2
Holokenmod	90	10	2083	3	2	5.2
Kerneladiutor	1347	21	1335	60	6	55.6
Koreader	7908	8104	2828	164	857	119.3
Languagepack	609	112	3147	31	189	1833.10
Lifecounter	2.2	17	2647	1	5	2.2
Lightning-browser	2125	1726	2879	72	744	23.10
Listmyaps	112	60	2670	2	21	2.3
Logmein-android	270	13	2394	7	11	1.10
Mlauncher	6	7	1968	1	2	0.2
Media-button-router	93	25	1949	1	6	1.3
Memento	82	142	1474	8	55	9.4
Memopad	61	7	3503	2	1	1.8
Openbikesharing	388	61	2327	28	52	4.10
Open-money-tracker	559	13	457	8	3	11.10
Openfoodfacts-androidapp	7218	576	2055	113	401	417.2
Openmw-android	862	192	1071	6	25	12.4
Permissionsmanager	54	4	1063	2	2	1.4
Pi-hole-droid	53	111	1400	4	15	147.6
Pixivformuzei3	870	90	603	11	11	5.0
Portauthority	1004	181	2200	14	53	4.10
Privacy-friendly-netmonitor	393	117	1510	14	30	8.1
Privacy-friendly-passwordgenerator	290	23	1497	4	12	8.3
Privacy-friendly-reckoning-skills	72	10	1326	5	2	4.9
Proexpense	276	40	188	3	12	16.2
Qbittorrent Client	930	211	2483	5	21481	29.0
Qrscan	96	25	1076	2	8	0.4
Rbb	2347	34	3150	1	4	27.6
search based launcher	257	40	2905	3	18	2.0
Smssync	1816	926	3608	21	468	42.7
Siteswap-generator	193	10	1174	2	4	8.2
Synctool	142	22	941	2	10	8.8
Tvhguide	364	44	3518	2	25	5.8
Taxiandroidopen	127	87	2573	1	115	8.0
Towercollector	561	97	1756	3	17	28.4
Trickytripper	301	43	3264	6	13	25.8
Ushahidi-android	949	205	4240	10	156	0.5
Vitosha-blackjack	16	7	1968	1	3	3.0
Voipms-sms-client	451	148	2283	4	49	12.9
Votar	65	14	2538	3	6	3.10
Weather	142	40	1753	7	13	22
Wulkanowy	1119	114	1351	23	18	100.2
Yashlang	161	23	473	1	1	26.10
Zeus	920	187	673	12	34	40.0

To further verify our classifier, we evaluated its performance on 5 randomly selected repositories from the set that was mined with the classifier. We perform keyword mining on these repositories in order to identify the false negatives of the classifier. Of the 5 repositories selected, 3 were found in both CM and KM to contain no performance NFP improving commits. In the repositories where commits were found, one was found to have 5 performance NFP improving commits compared to 3 found by the classifier, and in the other the same 3 commits were found by both approaches. These repositories are all small yet representative of many of the repositories which were mined. In order to evaluate the classifier on a larger repository with many commits, we also run it on the Koreader repository where the most CM commits were found (147 overall, see Table 7). We manually analyse all commits found using keyword search. In this project we found 2 additional relevant execution time commits, 2 additional memory commits, 1 additional frame rate improving commit and the same set of bandwidth improving commits with the keyword search. This means that the classifier only missed 5 relevant commits.

Our final manually curated corpus of performance NFP commits thus contains a total of 560 commits, which we use to answer our RQs.

### Step 3: Categorisation of Mined Performance NFP Improving Commits

To gain a greater understanding of the set of commits, we manually classified them by the type of change that was made. For each performance NFP (i.e., execution time, memory consumption, bandwidth, and frame rate) the set of extracted commits was examined and categories were generated, based on commit type. Commits were inserted into relevant categories or into new categories if they could not be classified inside current ones. Commits which could be classified into more than one category due to multiple changes were added to both categories. If two categories had a large shared membership or it became difficult to place a commit into either category, the categories were combined into a single category encompassing the traits of both.

Some commits were unclassifiable, e.g., due to improvements being buried in a large list of changes, or changes requiring domain specific knowledge that is not explained in the commit message. If many commits were left without a category, the uncategorised commits were re-examined to determine if any categories had not been uncovered in the first class. Next another author examined the categorised commits to analyse whether or not they belonged to a given category. In case of disagreement the commit was placed in the unknown category, this occurred in 15 instances. The two authors also independently examined the issues associated with these commits in order to gather any extra information that could aid us in categorising them. In the case of non-classifiable commits we had to rely on the description of the optimisation written by the developer in the commit message as evidence that there was an improvement.^9^

**Table 6:** Comparison between categories identified by keyword search vs. classifier. Percentages from total cumulate to > 100% as some commits address multiple NFP

	Keyword	Classifier	Discrepancy
Total	229 (100%)	331(100%)	–
Execution Time	125 (54.5%)	211(64.2%)	9.7%
Memory Usage	73 (31.9%)	115(34.3%)	2.3%
Bandwidth	26 (11.4%)	5 (1.5%)	9.9%
Framerate	15 (6.6%)	15 (4.6%)	2%

Table 6 summarizes the categories of the two separate datasets of found commits via keyword and classifier search respectively. The results show only around two percent discrepancy in the memory usage and framerate categories. The Execution time is represented 9.7% stronger via the classifier, and the bandwith around 9.9% less via the classifier. The reason for this may be a slight bias towards run time performance, but may also be simply because bandwidth is not as relevant for many projects. 19 out of 26 bandwidth commits we identified via keyword search are only from two repositories (see Table 7), and thus may simply be overrepresented in the keyword dataset. The analysis implies that the classifier can produce datasets that reflect the real-world considerations of Android developers towards NFP.

**Table 7:** RQ1: Number of NFP improving commits in each repository (% of total commits in repository). Repositories with zero NFP-improving commits are not listed. The “Total NFP Commits” column does not count duplicates (as some commits could have improved multiple properties at once)

App name	Execution time	Memory	Bandwidth	Frame rate	Total NFP
	commits	commits	commits	commits	commits
Android CUPS Print	1 (0.36%)	1 (0.36%)	1 (0.36%)	0	3 (1.09%)
Apple Flinger	1 (0.22%)	0	0	0	1 (0.22%)
Calculator	11 (0.96%)	1 (0.09%)	0	1 (0.09%)	13 (1.14%)
Call Recorder	0	2 (0.13%)	0	1 (0.06%)	3 (0.19%)
DNS66	4 (1.17%)	8 (2.34%)	1 (0.29%)	0	10 (2.93%)
F-Droid	56 (0.89%)	15 (0.24%)	9 (0.15%)	6 (0.10%)	85 (1.38%)
Firefox	10 (0.35%)	4 (0.15%)	2 (0.08%)	2 (0.08%)	18(0.66%)
Frozen Bubble	1 (0.64%)	3 (1.91%)	1 (0.64%)	0	3(1.91%)
G-Droid	2 (0.32%)	0	2 (0.32%)	0	3(0.48%)
Gadgetbridge	9 (0.17%)	17 (0.33%)	0	2 (0.04%)	28 (0.62%)
Mi Mangu Nu	6 (0.33%)	6 (0.33%)	0	0	12 (0.66%)
NewPipe	24 (0.41%)	16 (0.26%)	10 (0.17%)	3 (0.05%)	50 (0.82%)
KM Total	125	73	26	15	229
Alwayson	9 (2.48%)	1 (0.27%)	0	1 (0.27%)	11 (3.03%)
Android-inventory-agent	1 (0.10%)	1 (0.10%)	0	0	2 (0.20%)
Android-usb-serial-monitor-lite	1 (2.0%)	0	0	0	1 (2.0%)
Anewjkuapp	5 (0.37%)	0	0	0	5 (0.37%)
Atmospherelogger	2 (3.07%)	0	0	0	2 (3.07%)
Audioanchor	1 (0.41%)	0	0	0	1 (0.41%)
Avare	15 (0.83%)	8 (0.55%)	0	2 (0.11%)	22 (1.34%)
Changedetection	2 (0.91%)	0	0	1 (0.45%)	3 (1.36%)
Cmus-android-remote	1 (2.22%)	0	0	1 (2.22%)	1 (2.22%)
Controlloid-client	3 (3.15%)	0	2 (2.10%)	0	3 (3.15%)
Easytoken	0	1 (0.98%)	0	0	1 (0.98%)
Easywatermark	1 (0.8%)	0	0	0	1 (0.8%)
Gigaget	2 (1.56%)	1 (0.78%)	0	0	3 (2.34%)
Glt-companion	6 (1.24%)	4 (0.82%)	0	0	10 (2.07%)
Http-shortcuts	2 (0.17%)	0	0	0	2 (0.17%)
Kerneladiutor	1 (0.07%)	5 (0.29%)	0	0	6 (0.37%)
Koreader	54 (0.67%)	23 (0.25%)	0	3 (0.03%)	71 (0.89%)
Lightning-browser	26 (1.22%)	21 (0.98%)	0	2 (0.09%)	45 (2.11%)
Listmyaps	1 (0.89%)	1 (0.89%)	0	1 (0.89%)	2 (1.78%)
Media-button-router	1 (1.07%)	0	0	0	1 (1.07%)
Open_money_tracker	1 (0.17%)	0	0	0	1 (0.17%)
Openbikesharing	0	1 (0.25%)	0	0	1 (0.25%)
Openfoodfacts-androidapp	6 (0.08%)	2 (0.02%)	1 (0.01%)	0	8 (0.11%)
Openmw-android	2 (0.23%)	1 (0.11%)	0	0	3 (0.34%)
Pixivformuzei3	10 (1.14%)	8 (0.91%)	0	1 (0.11%)	17 (1.95%)
Portauthority	27 (2.68%)	25 (2.39%)	2 (0.19%)	1 (0.09%)	50 (4.88%)
Privacy-friendly-reckoning-skills	1 (1.38%)	0	0	0	1 (1.38%)
Proexpense	0	2 (0.72%)	0	1 (0.36%)	3 (1.08%)
Qbittorrent-client	2 (0.21%)	0	0	0	2 (0.21%)
Rbb	4 (0.17%)	2 (0.08%)	0	0	6 (0.25%)
Search-based-launcher-v2	1 (0.38%)	0	0	0	1 (0.38%)
Siteswap_generator	1 (0.51%)	0	0	0	1 (0.51%)
Smssync	2 (0.11%)	2 (0.11%)	0	1 (0.05%)	3 (0.16%)
Towercollector	6 (1.06%)	2 (0.35%)	0	0	8 (1.42%)
Trickytripper	1 (0.33%)	0	0	0	1 (0.33%)
Tvhguide	3 (0.82%)	2 (0.54%)	0	0	5 (1.37%)
Ushahidi_android	4 (0.31%)	1 (0.10%)	0	0	5 (0.42%)
Voipms-sms-client	4 (0.88%)	0	0	0	4 (0.88%)
Weather	1 (0.70%)	0	0	0	1 (0.70%)
Wulkanowy	2 (0.17%)	2 (0.08%)	0	0	4 (0.26%)
CM Total	211	113	5	15	331
Total	346	188	31	30	560

## Results

In this section we present the results of our mining for commits improving execution time, memory consumption, bandwidth usage, and frame rate. We report on these non-functional property improving (NFP) commits returned from our keyword search (KM) and those returned using our classifier (CM) separately. We investigate how developers improve the four NFPs, and categorise them to see whether source-code level changes could be implemented in automated software improvement tooling, targeting the mobile domain.

### RQ1: Numbers of NFP-Improving Commits Found

Table 7 shows the number of commits which were found to improve each particular NFP in each of the repository mined, split between KM and CM commits. We also report on what percentages of total commits improve each of the four NFPs considered.

**KM commits:** We found NFP-improving commits in 12 out of 20 most popular Android repositories. 229 out of a total of 28,028 were deemed to improve 1 of our four NFPs considered. Execution time is the most commonly improved non-functional property in our KM set of commits (with 125 commits identified), appearing to be the most important non-functional property to Android developers. The next most common improvement was memory usage, with 73 commits. In three repositories, the number of commits improving memory consumption was actually greater than those improving execution time, showing its importance varies across projects. Bandwidth is improved less often than the previous two properties. It is to be expected, as some applications use little to no network data. A lot of network traffic also consists of large files, such as videos, pictures, or application APKs. Decreasing the network data used by these files is often not possible with source code changes. Frame rate is not improved very often. This could be due to developers being willing to tolerate frame rate in their applications, or that changes which improve frame rate are less well-known amongst developers. Also we found that the changes are larger than those for memory and execution time, so may require more effort to implement (see Table 10).
**CM commits:** We found NFPs in 40 out of 80 randomly selected repositories. We find that our automatic classifier mostly selected performance improving commits (211). The next most commonly captured improved property was memory consumption with 115 out of 331 CM commits. Bandwidth and frame rate improving commits were, as with KM, much less common. With 15 frame rate commits and only 5 bandwidth improving commits found.

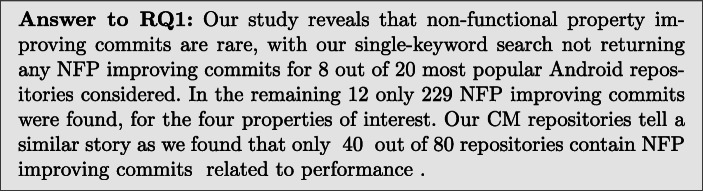


### RQ2: How Android Developers Improve NFPs

To answer RQ2 we first analyse general characteristics of the commits found, i.e., age of commits, functionality changes made at the same time as NFP improving changes, commit size, multiple NFPs improved at once, and optimisation trade-offs between NFPs. This should give us an overview of the current software development practice with respect to NFP improvement.

#### Age of Repository When Commits are Made

To determine when developers make commits that improve performance NFPs, we look at the age of the repository when a commit was made in days, i.e., how long after the first commit was it made (see Table 8). In this table we show the median number of days between the first commit and the commits of each category, we also show the upper and lower quartiles to show the spread of the ages. We find that the age of the repository when these commits are made varies greatly between repositories. Not only that, but the spread of this figure also varies. For example, in the F-Droid repository (highlighted in the table), NFP improving commits were spread out in age, even more so than other commits. Perhaps making NFP commits throughout the life cycle of the application rather than in small time windows is one of the reasons F-Droid was found to have the most NFP improving commits.

**Table 8:** RQ2: age in days of repositories when commits were made (CM repositories are highlighted in italic)

Repository	Execution time commits			Memory commits			Band. commits			Frame. commits			Other commits		
	LQ.	Med.	UQ.	LQ.	Med.	UQ.	LQ.	Med.	UQ.	LQ.	Med.	UQ.	LQ.	Med.	UQ.
Android CUPS Printer	1210	1210	1210	1206	1206	1206	1264	1264	1264	–	–	–	410	518	708
Apple Flinger	18	18	18	–	–	–	–	–	–	–	–	–	26	379	411
Calculator	1789	2000	2396	2444	2444	2444	–	–	–	1445	1445	1445	1092	1568	2003
Call Recorder	–	–	–	594	844	1095	–	–	–	–	–	–	212	478	698
DNS66	99	132	142	131	134	166	3	3	3	–	–	–	10	166	250
**F-Droid**	**1179**	**2047**	**2472**	**1150**	**1875**	**2724**	**1637**	**1778**	**1992**	**1744**	**2158**	**2380**	**1430**	**1949**	**2431**
Firefox	172	276	350	59	103	212	394	465	536	117	117	117	187	363	606
Frozen Bubble	1642	1642	1642	1505	1642	1654	1642	1642	1642	–	–	–	1282	1638	2270
G-Droid	87	98	110	–	–	–	122	122	122	–	–	–	45	76	124
Gadgetbridge	688	1222	1448	427	688	723	–	–	–	376	525	674	479	868	1406
Mi Mangu Nu	444	620	732	480	616	824	–	–	–	–	–	–	371	674	1154
NewPipe	819	1277	1440	879	903	1271	1204	1374	1482	760	785	900	614	947	1360
*Alwayson*	209	719	739	763	763	763	–	–	–	716	716	716	410	668	727
*Android-inventory-agent*	92	92	92	2847	2847	2847	–	–	–	–	–	–	2388	2459	2788
*Android-usb-serial-monitor-lite*	28	28	28	–	–	–	–	–	–	–	–	–	16	26	72
*Anewjkuapp*	403	1177	1200	–	–	–	–	–	–	–	–	–	221	543	1604
*Atmospherelogger*	132	134	135	–	–	–	–	–	–	–	–	–	131	439	2669
*Audioanchor*	533	533	533	–	–	–	–	–	–	–	–	–	130	336	533
*Avare*	584	1003	1493	287	1135	1274	–	–	–	393	517	641	387	763	1319
*Changedetection*	35	47	59	–	–	–	–	–	–	130	130	130	23	40	126
*Cmus-android-remote*	8	8	8	–	–	–	–	–	–	8	8	8	3	5	9
*Controlloid-client*	54	54	82	–	–	–	54	54	54	–	–	–	13	50	115
*Easytoken*	–	–	–	34	34	34	–	–	–	–	–	–	7	11	13
*Easywatermark*	57	57	57	–	–	–	–	–	–	–	–	–	23	37	44
*Gigaget*	11	12	14	27	27	27	–	–	–	–	–	–	8	15	83
*Glt-companion*	462	928	1026	894	974	1060	–	–	–	–	–	–	357	979	1519
*Http-shortcuts*	251	272	294	–	–	–	–	–	–	–	–	–	653	1121	1816
*Kerneladiutor*	594	594	594	459	590	613	–	–	–	–	–	–	114	301	597
*Koreader*	727	1402	2314	933	1727	2289	–	–	–	1381	1580	2420	617	1115	2303
*Lightning-browser*	554	954	1704	1059	1104	1202	–	–	–	318	584	849	800	1310	1725
*Listmyaps*	248	248	248	248	248	248	–	–	–	2	2	2	24	31	186
*Media-button-router*	332	332	332	–	–	–	–	–	–	–	–	–	6	16	262
*Open-money-tracker*	601	601	601	–	–	–	–	–	–	–	–	–	530	602	1193
*Openbikesharing*	–	–	–	193	193	193	–	–	–	–	–	–	83	200	541
*Openfoodfacts-androidapp*	1218	1648	1813	919	1230	1540	1859	1859	1859	–	–	–	717	1003	1427
*Openmw-android*	544	793	1042	1827	1827	1827	–	–	–	–	–	–	321	1292	1717
*Pixivformuzei3*	162	274	364	85	89	92	–	–	–	393	393	393	119	273	383
*Portauthority*	226	601	794	647	739	791	1088	1146	1205	615	615	615	273	676	831
*Privacy-friendly-reckoning-skills*	277	277	277	–	–	–	–	–	–	–	–	–	78	110	277
*Proexpense*	–	–	–	174	175	176	–	–	–	167	167	167	20	43	150
*Qbittorrent-client*	227	246	264	–	–	–	–	–	–	–	–	–	394	615	960
*Rbb*	122	267	433	127	222	317	–	–	–	–	–	–	216	350	543
*Search-based-launcher-v2*	939	939	939	–	–	–	–	–	–	–	–	–	952	988	1014
*Siteswap-generator*	10	10	10	–	–	–	–	–	–	–	–	–	46	197	397
*Smssync*	1380	1578	1775	1293	1520	1746	–	–	–	1182	1182	1182	1135	1551	1837
*Towercollector*	14	34	167	528	886	1244	–	–	–	–	–	–	674	972	1447
*Trickytripper*	611	611	611	–	–	–	–	–	–	–	–	–	445	1088	1499
*Tvhguide*	37	52	173	227	386	545	–	–	–	–	–	–	23	69	210
*Ushahidi-android*	663	683	915	667	667	667	–	–	–	–	–	–	710	974	1417
*Voipms-sms-client*	724	726	960	–	–	–	–	–	–	–	–	–	694	1002	1667
*Weather*	209	209	209	–	–	–	–	–	–	–	–	–	107	166	1047
*Wulkanowy*	598	796	993	275	275	275	–	–	–	–	–	–	702	946	1174

#### Functionality Changes in Commits

Table 9 shows the number of commits of each type in which functional changes were also made. For the KM commits three of the types contained very similar numbers (14%), however bandwidth improving commits contained more (30%). This is mostly due to many of the bandwidth commits coming from repositories in which commits tended to be larger and contain many changes. The CM commits are similar, with the exception of frame rate, where 1/3 of commits also modified functionality.

**Table 9:** RQ2: Number of commits changing both functional and non-functional properties

Type of commit	No. of commits	
	KM	CM
Exec. Time Commits	17 (14%)	24 (11%)
Memory Commits	10 (14%)	10 (9%)
Bandwidth Commits	8 (30%)	1 (20%)
Frame Rate Commits	2 (13%)	5 (33%)

#### Size of Commits

To determine the size of non-functional property improving commits we consider four measures: the number of files changed, the number of chunks changed, the number of classes modified, and the number of lines of code inserted/removed. Git splits the diff of each commit into chunks, where a chunk is the set of lines containing a change and the surrounding lines. If changes are close together they will be contained within the same chunks, so chunks can be used to measure the distribution of changes. These measures will provide a holistic picture of both the size of commits and the distribution of the changes that are made. Some of the commits contained multiple changes which will inflate their size, however so do many standard commits. We also take median values to attempt to mitigate this distortion and allow for a valid comparison. We compare the size of our identified performance NFP improving commits to the size of every other commit found in the repositories. As commits from both categories will contain multiple changes, we do not attempt to distinguish between functional changes and non-functional changes in individual commits.

As shown in Table 10 we can see that KM commits improving performance NFPs tend to be larger than a generic commit, in every measure. They often span multiple files, multiple chunks and change multiple class definitions. They also add in more lines than they remove. For the CM commits, execution time and memory improving commits are of similar size to those in the KM set, spanning multiple files and make larger changes than other commits. The CM bandwidth commits are smaller than those in KM set, however still make larger changes than non NFP improving commits. Finally, the CM frame rate improving commits are very large (median lines inserted of 47 for KM and 77 for CM). This could be due to the large rate of frame improving commits which also alter the functionality of their projects, as shown in Table 9.

**Table 10:** RQ2: Median commit sizes. ‘Other’ category represents all commits that were not deemed to improve any of the four NFPs of interest

Type of commit	Files changed		Chunks changed		Classes changed		Lines inserted		Lines removed	
	KM	CM	KM	CM	KM	CM	KM	CM	KM	CM
Exec. Time	2	2	5	5	4	2	16	9	12	6
Memory	1	2	4	4	3	2	16	7	6	5
Bandwidth	3	1	13	1	10	1	51	10	13	1
Frame Rate	1	3.0	9	16	7	4.5	47	77	4	4
Other	1	1	3	2	1	1	6	4	2	1

#### Multiple Improvements

Table 11 shows how often commits improve multiple properties at the same time. The vast majority of NFP improving commits improve one property at a time. This is true across both CM and KM commits. However, in 6.5% of cases (5.7% of KM and 7.0% of CM) developers are able to improve multiple properties at once. It is possible that some commits do improve multiple properties in ways that developers are not aware of or do not report, as this is not the primary purpose of the commit.

**Table 11:** RQ2: Commits improving multiple properties

	Execution time		Memory		Bandwidth		Frame rate	
	KM	CM	KM	CM	KM	CM	KM	CM
Exec. Time	**125**	**211**	6	15	5	5	0	5
Memory	6	15	**73**	**115**	2	0	0	3
Bandwidth	5	5	2	0	**26**	**5**	0	0
Frame Rate	0	5	0	3	0	0	**15**	**15**

Values in boldface indicate that the two properties being improved are the same

#### Tradeoffs

Some commit messages report that changes which improve one non-functional property negatively affect others. Table 12 shows that memory and execution time improvements are often traded-off against each other. The most often impaired property is memory. This is due to the use of caching. Caching can be used to avoid having to repeatedly call the same code by storing the result. If the code not being called accesses the network, caching can reduce bandwidth usage. Sometimes caches can be too big so their size must be reduced. This can have a negative impact on execution time. 
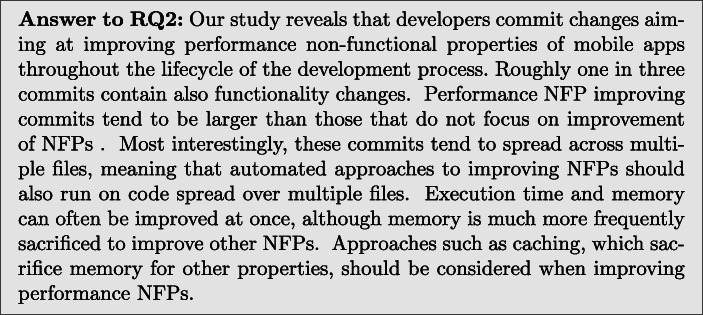

**Table 12:** RQ2: Commits with trade-offs between properties

	Exec. time		Memory			Frame rate		
	KM	CM	KM	CM	KM	CM	KM	CM
Execution Time	**0**	**0**	20	25	0	0	0	0
Memory	6	2	**0**	**0**	0	0	0	0
Bandwidth	0	0	9	0	**0**	**0**	0	0
Frame Rate	0	0	0	0	0	0	**0**	**0**

Values in boldface indicate a trade-off between the same category

### RQ3: Types of NFP Commits

Subsequently, we discuss the results of the manual categorisation of NFP improving commits, as explained in Section 2.5. This should help us establish whether changes made by developers are already automated by existing tooling, and if not, whether new refactorings could be suggested for future work.

**Fig. 1:**
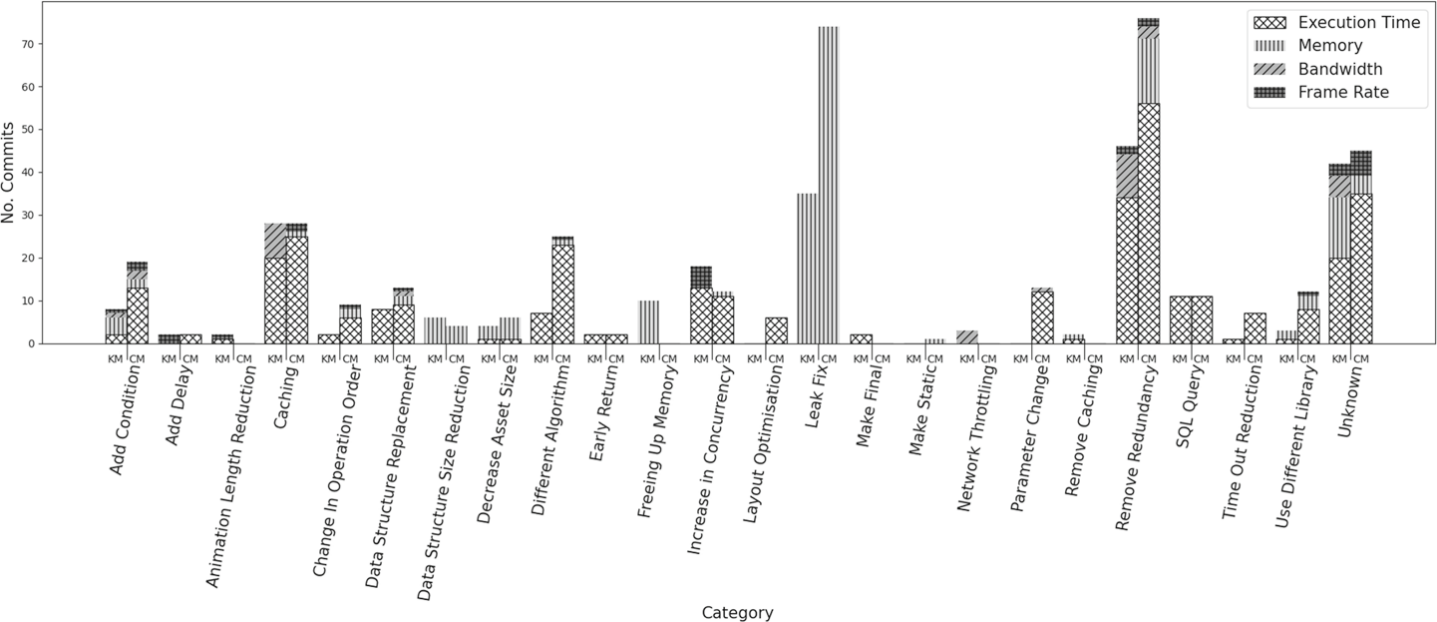
Histogram showing the distribution of commits amongst different categories

In order to answer RQ3, our categorisation is presented in Table 13, we also show this data in Fig. 1 as a histogram for easy comparison.

**Table 13:** RQ3: Categories of commits by non-functional property (% of commits improving a particular NFP)

Category	Subcategory	Execution time		Memory		Bandwidth		Frame rate	
		KM	CM	KM	CM	KM	CM	KM	CM
Add Condition	–	2 (1.6%)	13 (6.2%)	4 (5.5%)	2 (1.8%)	1 (3.8%)	2 (40.0%)	1 (6.7%)	2 (13.3%)
Add Delay	–	0	2 (< 1%)	0	0	0	0	2 (13.3%)	0
Animation Length Reduction	–	1 (< 1%)	0	0	0	0	0	1 (6.7%)	0
Caching	–	20 (16.0%)	25 (11.8%)	0	1 (< 1%)	8 (30.8%)	0	0	2 (13.3%)
Change In Operation Order	–	2 (1.6%)	6 (2.8%)	0	2 (1.8%)	0	0	0	1 (6.7%)
Data Structure Replacement	–	8 (6.4%)	9 (4.3%)	0	2 (1.8%)	0	1 (20.0%)	0	1 (6.7%)
Data Structure Size Reduction	–	0	0	6 (8.2%)	4 (3.5%)	0	0	0	0
Decrease Asset Size	–	1 (< 1%)	1 (< 1%)	3 (4.1%)	5 (4.4%)	0	0	0	0
Different Algorithm	Use String Builder	0	4 (1.9%)	0	0	0	0	0	0
	Use Char instead of String	0	3 (1.4%)	0	0	0	0	0	0
	Improve Regex Performance	2 (1.6%)	4 (1.9%)	0	0	0	0	0	0
	Other	5 (4.0%)	12 (5.7%)	0	1 (< 1%)	0	0	0	1 (6.7%)
Early Return	–	2 (1.6%)	2 (< 1%)	0	0	0	0	0	0
Freeing Up Memory	–	0	0	10 (13.7%)	0	0	0	0	0
Increase in Concurrency	Move code to background	7 (5.6%)	9 (4.3%)	1 (1.4%)	1 (< 1%)	0	0	5 (33.3%)	0
	Alter timing	3 (2.4%)	0	0	0	0	0	0	0
	Use a thread pool	0	2 (< 1%)	0	0	0	0	0	0
Layout Optimisation	–	0	6 (2.8%)	0	0	0	0	0	0
Leak Fix	–	0	0	35 (47.9%)	74 (65.5%)	0	0	0	0
Make Final	–	2 (1.6%)	0	0	0	0	0	0	0
Make Static	–	0	0	0	1 (< 1%)	0	0	0	0
Network Throttling	–	0	0	0	0	3 (11.5%)	0	0	0
Parameter Change	–	0	12 (5.7%)	0	0	0	1 (20.0%)	0	0
Remove Caching	–	1 (< 1%)	0	1 (< 1%)	0	0	0	0	0
Remove Redundancy	–	34 (27.2%)	56 (26.5%)	0	15 (13.3%)	10 (38.5%)	3 (60.0%)	2 (13.3%)	2 (13.3%)
SQL Query	Change Primary Key	0	2 (< 1%)	0	0	0	0	0	0
	Specify column	0	2 (< 1%)	0	0	0	0	0	0
	Combine Queries	0	3 (1.4%)	0	0	0	0	0	0
	Move file into Database	1 (< 1%)	2 (< 1%)	0	0	0	0	0	0
	Remove unneeded JOIN	1 (< 1%)	0	0	0	0	0	0	0
	Flatten queries	3 (2.4%)	0	0	0	0	0	0	0
	Add table indices	3 (2.4%)	0	0	0	0	0	0	0
	Use transactions	1 (< 1%)	1 (< 1%)	0	0	0	0	0	0
	Parameter Binding	0	1 (< 1%)	0	0	0	0	0	0
Time Out Reduction	–	1 (< 1%)	7 (3.3%)	0	0	0	0	0	0
Use Different Library	–	1 (< 1%)	8 (3.8%)	2 (2.7%)	3 (2.7%)	0	0	0	1 (6.7%)
Unknown	–	20 (16.0%)	35 (16.6%)	14 (19.2%)	4 (3.5%)	5 (19.2%)	0	5 (33.3%)	6 (40.0%)

**Fig. 2:**
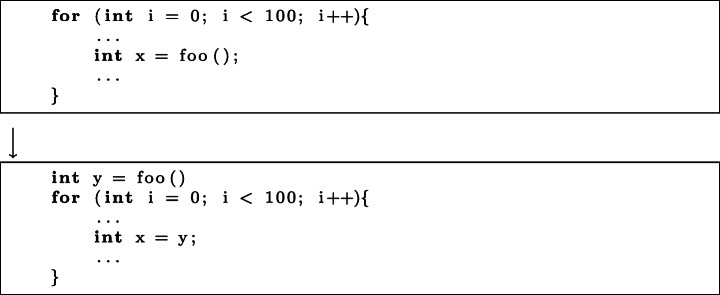
An example of the caching pattern

**Add Condition:** Conditions were added in 8 KM and 19 CM commits, this allowed applications to avoid additional computation unless it was actually necessary.
  *Execution Time:* 2 KM and 13 CM commits improved execution time by using caching. This is the most obvious application of caching as one can use it to avoid repeatedly performing the same computation unnecessarily.
  *Memory:* 4 KM commits and 2 CM commits optimised memory usage by adding new conditions.
  *Bandwidth:* 1 KM and 2 CM commits utilised caching to avoid making unnecessary network requests.
  *Frame Rate:* 1 KM and 2 CM commits utilised new conditions to avoid unnecessary work on the UI thread, thus improving frame rate.
  *Pattern:* Wrap blocks of code in if statements, or add new conditions to existing if statements.
**Add Delay:** Delays were introduced in 2 KM and 2 CM commits, these delays allowed background execution to finish before proceeding, thus improving performance NFPs.
  *Execution Time:* 2 CM commits improved execution time using increased delays.
  *Frame Rate:* 2 KM commits utilised delays to improve frame rate.
  *Pattern:* Insert calls to the Time.sleep() method into code.
**Animation Length Reduction.**: Visual changes, such as changes to animations or UI elements, are used in 2 KM commits to reduce the frame rate of an application.
  *Frame Rate:* 1 KM commit animation length reduction improved the application execution time.
  *Frame Rate:* 1 KM commit used programmatic animation changes were used to improve the application frame rate.
  *Pattern:* When animations are done programmatically we recommend using profilers to identify hotspots. Frames could also be removed from animations to speed them up and a trade off between speed and smoothness could be found.
**Caching.**: Caching data to avoid rerunning code was one of the largest categories of change, with 28 KM and 28 CM changes being made.
  *Execution Time:* 20 KM and 25 CM commits improved execution time using caching. This is the most obvious application of caching as we one use it to avoid repeatedly performing the same computation unnecessarily.
  *Memory:* Whilst caching can often increase memory usage, if used to avoid memory intensive computation it can actually save memory. However, this is uncommon, with only 1 CM commit showing this use of caching.
  *Bandwidth:* 8 KM commits utilised caching to avoid making unnecessary network requests.
  *Frame Rate:* 1 CM commit utilised caching to avoid making unnecessary work on the UI thread, improving frame rate.
  *Pattern:* Caching is a common pattern for decreasing the execution time of an application. It can often be easily implemented by assigning the results of a method call to a variable and replacing future calls to the method with that variable. An example of a caching pattern being applied can be found in Fig. 2.

**Fig. 3:**
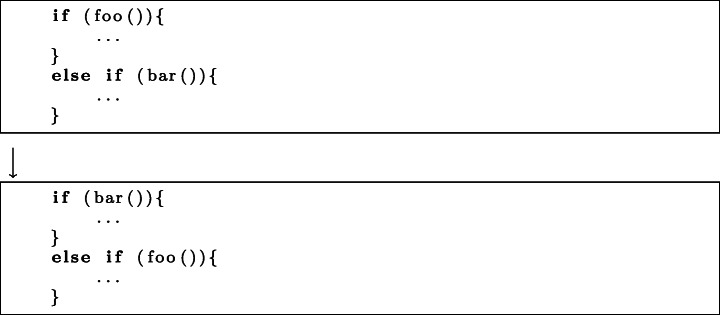
An example of the change in operation order pattern

**Change in Operation Order.**: 2 KM and 9 CM commits altered the order of operations. This involved swapping the order in which lines of code are executed, such as conditions in if statements.
  *Execution Time:* 2 KM and 6 CM commits improved the execution time of code by changing the order of operations.
  *Memory:* 2 CM commits were found to reduce the memory consumed.
  *Frame Rate:* only 1 CM commit changed the order of operations in order to improve the application frame rate.
  *Pattern:* Change in operation order can be achieved by swapping lines or blocks of code, or nodes in the abstract syntax tree. The swap operator has already been utilised in program repair (Le Goues et al. 2012), but is yet to be widely adopted in automated search-based techniques for improvement of NFPs of software (Petke et al. 2018). An example of this pattern can be seen in Fig. 3.

**Fig. 4:**
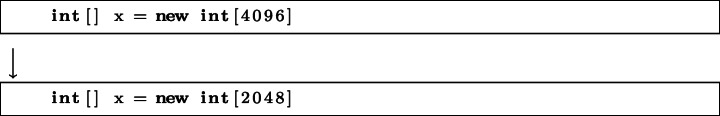
Data structure size reduction pattern

**Data Structure Replacement.**: Data structure replacements were used in 8 KM and 12 CM commits. A data structure replacement could consist of swapping an ArrayList for a LinkedList where the LinkedList is more efficient.
  *Execution Time:* 8 KM and 9 CM commits replaced data structure to improve execution time.
  *Memory:* 2 CM commits implemented the usage of more memory efficient data structures.
  *Bandwidth:* 1 CM commit replaced a data structure transmitted over the network with a smaller equivalent to improve bandwidth usage.
  *Frame Rate:* 1 CM commit used a more efficient data structure to improve frame rate.
  *Pattern:* Various NFPs can be improved by finding data structures and automatically replacing them with compatible ones. Similar approach has been implemented by Basios et al. (2018), although their tool is not publicly available.
**Data Structure Size Reduction.**: Reduction in the size of data structures, such as arrays, were used in 6 KM and 4 CM commits.
  *Memory:* All KM and CM commits improved the memory consumption of applications by reducing the sizes of data structures.
  *Pattern:* Reduce the size of data structures. This can be achieved, for instance, by changing the size of declared arrays. Program analysis could be required to prevent overflows. An example of this pattern can be seen in Fig. 4.

**Fig. 5:**
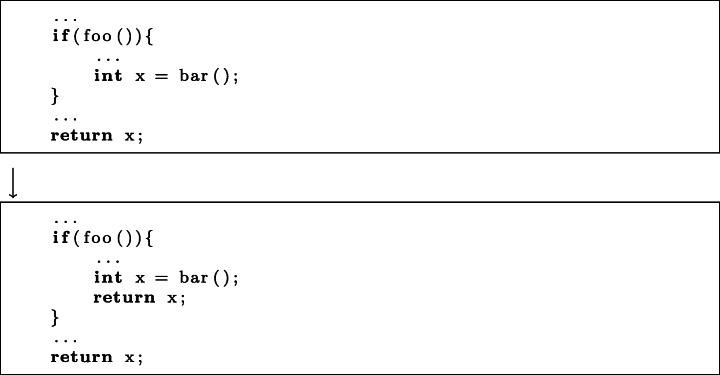
An example of the early return pattern

**Decrease Asset Size.**: Changes to assets such as images and fonts, mostly to improve the efficiency of loading said assets, account for 3 KM changes and 5 CM changes.
  *Execution Time:* 1 KM and 1 CM commit improved the execution by reducing asset size and speeding up their loading.
  *Memory:* 3 KM commits and 5 CM commits improved memory by reducing the amount of memory which large assets consume.
  *Pattern:* Use compression algorithms, such as gzip, to reduce the size of assets.
**Different Algorithm.**: The implementation of more efficient algorithms constituted 7 KM commits and 24 CM commits.
  *Execution Time:* 7 KM and 23 CM commits implemented more time efficient versions of algorithms.
  *Memory:* 1 CM commit replaced one algorithm with another more memory efficient one.
  *Frame Rate:* 1 CM commit implemented a more efficient algorithm to improve frame rate.
  *Subcategories:*
  **Use String Builder:** In 4 CM commits, String Builders were used in place of naive string construction to improve execution time.
  **Use char instead of String:** In 3 CM commits, method calls with Strings for arguments, such as indexOf, were replaced with equivalent methods with char arguments to improve execution time.
  **Improve Regex Performance:** In 2 KM and 4 CM commits, regular expressions were modified to execute more quickly.
  **Other:** 5 KM and 12 CM commits consisted of changes to algorithms which could not be grouped with others. We detail each of these changes in Appendix B.
**Early Return.**: Earlier return statements were introduced in 2 KM and 2 CM commits, preventing whole methods from executing when unnecessary.
  *Execution Time:* 2 KM and 2 CM commits used earlier returns to speed up application execution time.
  *Pattern:* Insertion of a return statement. An example of this pattern can be found in Fig. 5.

**Freeing Up Memory.**: Adjustments to the amount of memory in use when devices were low on memory was another common change made to 10 KM commits.
  *Memory:* All commits which used this strategy were used to reduce the memory consumption of applications.
  *Pattern:* Program analysis would be required to identify which resources could be freed from memory.
**Increase in Concurrency**: Multi-threading changes were used in 15 of the KM and 12 of the CM changes.
  *Execution Time:* 10 KM and 11 CM commits used more multi threading to speed up applications.
  *Memory:* 1 CM commit used more concurrency to become more memory efficient.
  *Frame Rate:* 5 KM commits improved frame rate by using multi-threading to reduce the load on the UI thread.
  *Subcategories:*
  **Move code to background:** 6 KM and 4 CM improved execution time, 1 CM commit improved memory consumption, and 3 KM commits improved frame rate by executing code on separate threads.
  **Alter timing:** 3 KM commits improved execution time by altering the timing of threads.
  **Use a thread pool:** 2 CM commits found improvements to execution time by introducing thread pools to manage thread execution more efficiently.
**Layout Optimisation.**: 6 CM commits modified the layouts of applications. This consists of flattening layouts to reduce nesting.
  *Execution Time:* All 6 CM commits were used to speed up applications.
  *Pattern:* Automatic layout flattening obtained by removing layout components and replacing them with their child components. This can be achieved with the lint tool.^10^
**Leak Fix**: Fixing memory leaks was the most common approach to decrease memory consumption, with 35 KM and 74 CM commits.
  *Memory:* Leak fixes were made in 35 KM commits, which is almost half of KM memory improving changes. 74 CM commits fixed leaks.
  *Pattern:* Automatically detecting objects that are being unnecessarily instantiated or not properly disposed of, using a tool like infer,^11^ and removing them with program repair techniques like GenProg (Le Goues et al. 2012).
**Make Final:** 2 KM commits introduced the final keyword to local variables, allowing more efficient code to be compiled.
  *Execution Time:* both commits improved execution time.
  *Pattern:* Add the static keyword to local variables.
**Make Static:** 1 CM commit added the static keyword to a method in order to reduce memory usage.
  *Memory:* The only commit in this category improved memory usage.
  *Pattern:* Add the static keyword to methods.
**Network Throttling.**: Network throttling (which is an intentional slowing down of internet speed) was used in 3 KM commits to improve the bandwidth usage. In fact, while this may not reduce the total amount of bandwidth used, it can still be useful to reduce the load on the network by speeding it up for other users.
  *Bandwidth:* All 3 KM commits used Network throttling to improve bandwidth usage.
  *Pattern:* Create a network monitor which reduces the networking of the application when traffic is high, a tool like android-varanus^12^ could be used to this end.
**Parameter Change.**: 13 CM commits simply changed parameters in various function calls, speeding up the application.
  *Execution Time:* 12 CM commits were used to decrease execution time.
  *Bandwidth:* 1 CM commit was used to improve frame rate.
  *Pattern:* Techniques such as deep parameter optimisation (Wu et al. 2015), have proven useful for finding optimal parameters in source code.
**Remove Caching:** 2 KM commit removed a cache to improve NFPs.
  *Execution Time:* 1 commit in this category improved execution time by removing a costly caching operation.
  *Memory:* 1 commit in this category improved memory consumption.
  *Pattern:* Replace cached variables with method calls.
**Remove Redundancy.**: The removal of redundant function calls or iterations is the largest category we identified, with 46 KM and 65 CM commits found. Removing unnecessary code can be an easy and simple way to optimise software.
  *Execution Time:* 34 KM and 56 CM changes improved execution time by removing unnecessary execution of code.
  *Memory:* 15 CM commits removed code instantiating objects, thus reducing memory consumption.
  *Bandwidth:* 10 KM and 3 CM changes removed unnecessary network access, reducing bandwidth usage.
  *Frame Rate:* 2 KM and 2 CM commits removed redundant code that was causing frame rate to be low.
  *Pattern:* Remove lines or blocks of code, or nodes in the abstract syntax tree. This operation is standard in Genetic Improvement (Petke et al. 2018) tooling used for automated improvement of non-functional software’s properties, such as execution time, energy consumption, but also for automated program repair.
**SQL Query.**: A large number of changes (11 KM and 11 CM) to SQL requests appear for improving performance NFPs. These changes were only present in 7 projects. Some of the changes removed unnecessary JOIN statements, others changed the order of JOIN statements.
  *Execution Time:* All SQL query commits improved execution time.
  *Subcategories*
  **Change Primary Key:** 2 CM commits improved execution times by changing the primary keys used in SQL tables.
  **Specify Column:** 2 CM commits moved from selecting all columns to selecting individual columns, speeding up query execution.
  **Combine Queries:** 3 CM commits combined multiple queries together to save executing them each individually.
  **Move File to DB:** 1 KM commit and 2 CM replaced file I/O with an in memory database to improve the execution time.
  **Remove Unneeded JOIN:** 1 KM commit improved execution time by removing an unnecessary JOIN statement.
  **Flatten Queries:** 3 KM commits flattened queries containing sub-queries into a single select query, improving execution time.
  **Add table indices:** 3 KM commits added indices to tables to speed up SQL queries.
  **Use transactions:** 1 KM commit and 1 CM commit wrapped a series of queries up into a single transaction, allowing them to be executed more quickly.
  **Parameter Binding:** 1 CM commit introduced parameter binding, which allows similar queries to be made repeatedly with only their parameters changed.
**Time Out Reduction.**: In total, 1 KM and 7 CM commits made changes to the length of timeouts, waiting for other computation to complete.
  *Execution Time:* 1 KM commit and 7 CM commits reduced unnecessarily long timeouts to improve the execution time.
  *Pattern:* Change timeout values in source code.
**Use Different Library.**: 3 KM 11 CM commits replaced the libraries they used with more efficient alternatives.
  *Execution Time:* 1 KM and 8 CM commits used different faster libraries.
  *Memory:* 2 KM and 3 CM commit used a more memory efficient library.
  *Frame Rate:* 1 CM commit used a more efficient library to improve frame rate.
  *Pattern:* Use a set of similar libraries and automatically replace their existing usages in code.
**Unknown.**: Some of the commits (41 KM and 46 CM) were not classifiable as the type of optimising change was not obvious from the commit message or the diff. In fact, some changes were bundled within large commits making optimisations hard to pinpoint, yet from the developer message it was clear that execution time, memory consumption, bandwidth, or frame rate had been improved.

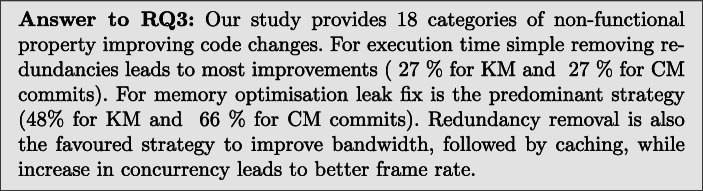


## Discussion

In this section we discuss potential avenues for future research that stem from our study.

### Recommendations for NFP Mining

Our work shows that a more fine-grained mining of NFP commits is feasible. The classifier that we presented has a good chance of finding commits with a recall of 0.8 (4 in 5 commits are found in our test set). The precision of 0.72 suggests that around 3 in 4 commits will be relevant. This means that the manual effort to identify relevant commits is much less than searching via keywords. This is confirmed by our mining via the classifier where we find 331 relevant commits by analyzing 495 commits. In comparison via keyword search we mined 229 relevant commits by manually checking 3,132 commits. I.e. analyzing 1.50 commits via classifier returns one relevant commit, vs. 13.68 commits for one relevant commit via keyword search.

This enables the mining of repositories for NFP commits on a larger scale than previously possible as the manual effort is reduced significantly. With larger datasets in the future it may even be possible to expand the classification to identify categories and subcategories of NFP that a commit falls under.

#### Characteristics of Repositories Containing NFP Improving Commits

As we observed that the number of performance NFP improving commits greatly varies among the mined repositories, we further analyse these repositories in an attempt to find the characteristics of those repositories that contain many commits. This can allow future NFP mining studies to target the most commit rich repositories.

Tables 1 and 5 show the properties (i.e., the total number of commits made in the repository, the number of stars a repository has, the number of developers who have contributed, the age of the repository, the number of times the repository has been forked, and the number of lines of code in the repository) of the repositories together with the number of performance NFP improving commits found in each of them.

In order to quantify the relationship between each property and the number of NFP improving commits found, we calculate the Pearson Correlation Coefficient (Pearson 1895). Pearson’s correlation (*ρ*) measures the linear relationship between two pairs of observations. It ranges from + 1 (indicating perfect correlation) to − 1 (indicating perfect inverse correlation), no correlation is indicated by 0. The results of this analysis are shown in Table 14.

**Table 14:** Correlation between properties of repositories and the number of NFP improving commits found in them

Property	Correlation coefficient (*ρ*)	*p* − *value*
Total commits	0.754	< 2.2*e* − 16
No. Stars	0.678	1.10*e* − 13
No. Contribs.	0.601	2.34*e* − 10
Age of Repo.	0.169	0.107
No. Forks.	0.043	0.697
KLoc	0.033	0.756

We find that the total number of commits in a repository shows the strongest, statistically significant, positive correlation with the number of performance NFP improving commits (*ρ* = 0.754, *p*-value < 2.20*e* − 16). We also find strong, statistically significant, positive correlations between the number of NFP improving commits and both the number of stars (*ρ* = 0.678, *p*-value = 1.10*e* − 13) and the number of contributors (*ρ* = 0.601, *p*-value = 234*e* − 10). We find no statistically significant correlation with the age of a repository (*ρ* = 0.169, *p*-value = 0.107), the number of lines of code in a repository (*ρ* = 0.043, *p*-value = 0.756), or the number of forks of the repository (*ρ* = 0.033, *p*-value = 0.697).

**Fig. 6:**
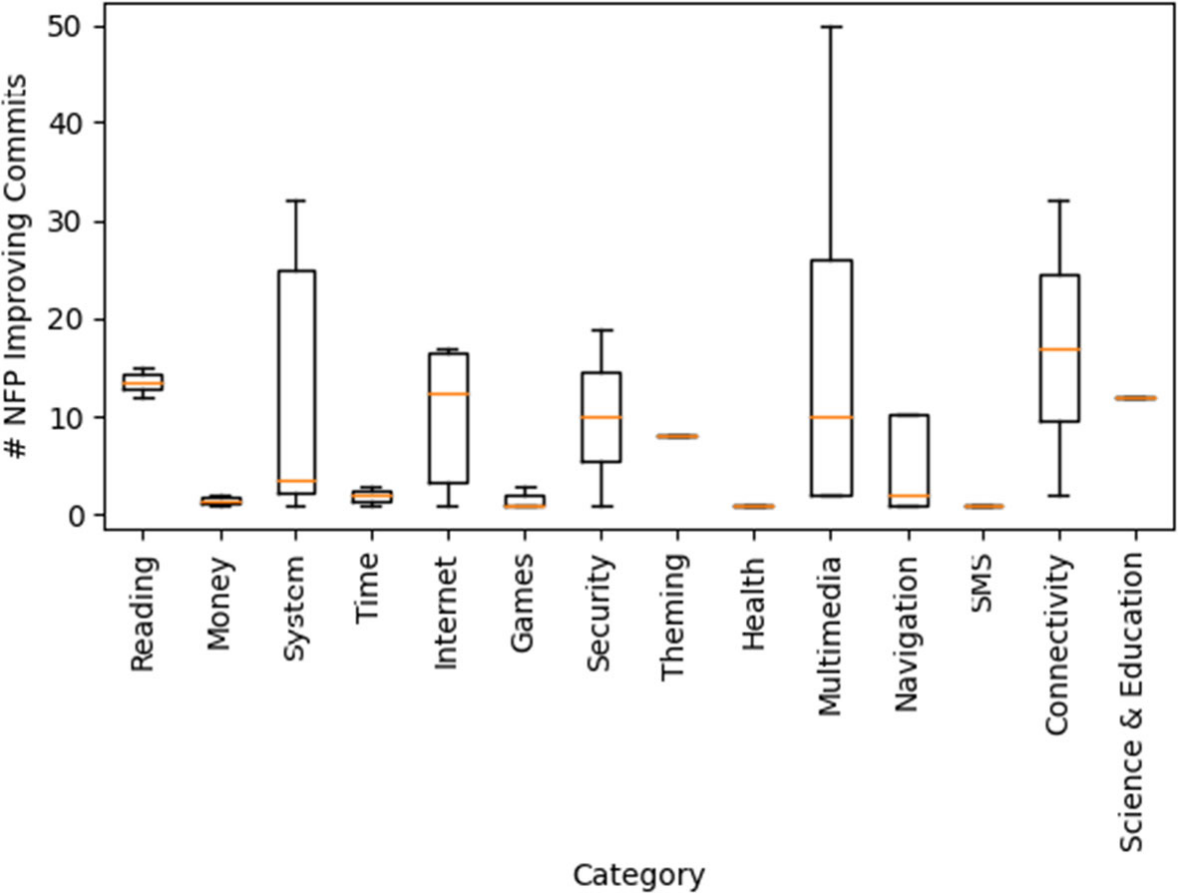
Box plot showing the relationship between repository category and number of performance NFP improving commits

We also attempt to see if there is a relationship between the categories of applications and the number of performance NFP improving commits we found. We present in Fig. 6 a box plot of the categories reported on F-Droid for each application against the number of performance NFP improving commits we found in it. We can observe that there are no categories that tend to have significantly more commits than others, however the applications in the connectivity category have the highest median number of NFP improving commits.

The above results suggest that it would be preferable to target large repositories that receive many stars by GitHub users and have a large number of contributors, when mining for performance NFPs commits.

### Recommendations for Performance NFP-Improving Tooling

Our analysis of commits improving execution time, memory consumption, bandwidth usage, and frame rate of mobile applications provides several findings. In particular, our main goal has been to establish how Android developers improve these four NFPs, and whether we could utilise this knowledge to enhance automated software improvement tooling.

After careful analysis of the literature, we found that 5 out of 23 improvement strategies (see Section 3) are already mimicked by software mutation operators in automated software improvement tooling, as described below.

Deletion of redundancy has been used in many fields, ranging from slicing to newer strategies such as genetic improvement (Petke et al. 2018). The *delete* operator, which can be applied at various granularity levels, e.g., line or statement level has proved effective in automated program repair (Goues et al. 2012). Similarly, 75% of the non-functional properties studied were improved through the removal of a redundancy (used to improve execution time, memory consumption, and frame rate). Android applications contain many lines of redundant code and its removal is one of the main techniques developers use to improve the four NFPs considered in this work.

Burles et al. (2015) and Basios et al. (2018) swap the data structures to improve non-functional properties. This is also reflected in our mined data. Both the *execution time* and *memory consumption* commit sets contained changes which consisted of simply swapping one data structure for a more efficient one. This suggests that patches produced with their techniques will be similar to those made by humans.

The *early return* category is small but offers a simple software transformation. Rather than assigning a value to a variable, just return the value. This will only be valid if the return value will not change in the rest of the method. This could be used as an opportunity to insert early returns. Recently, Brownlee et al. (2020) incorporated this type of operator in their automated software improvement tooling for Java software.

Switching the order of operations in which these are executed is also mimicked by the *swap* operator in genetic improvement research. Such swaps have been done at the statement (Brownlee et al. 2019) and expression level (Schulte et al. 2014; Haraldsson et al. 2017).

The *parameter change* category is reminiscent of deep parameter optimisation (Wu et al. 2015), where parameters are exposed and automatically tuned to improve some property. Bokhari et al. (2017) successfully applied deep parameter optimisation to energy consumption in Android. Our results suggest that deep parameter optimisation could be effective for improving other properties in Android.

Although the changes mentioned above have been incorporated into automated software improvement tooling, these have generally not been applied in the Android ecosystem, but just to traditional software.

Our study has also revealed several improvement strategies that are not yet employed in current search-based software improvement tools.

One such strategy is caching. Caching has been repeatedly used to improve execution time, memory consumption, and bandwidth usage. Caching is useful as it reduces calls to areas of code by storing their output. The stored value is then reused instead of calling the code. However, it does increase memory usage. The trade-off between the NFPs that caching improves must be balanced with the cost to memory, pointing to multi-objective optimisation. A caching pattern could be as simple as replacing multiple calls to a function, with the same arguments, with a single call to the function. The result of this call should be stored in a variable, and the original calls to the function replaced by the variable. Another option would be to cache a function called in a loop, replacing the function call in the loop with a variable storing the result of the function which is called before the loop, as in Fig. 2.

Changes to assets, such as pictures, are found among the commits that improve execution time and memory consumption. Assets can be resized to improve memory consumption (and potentially bandwidth usage) or handled differently to improve execution time. When modifying assets, the changes made must result in the asset still being acceptable to the users. Measuring this acceptability and finding reasonable modifications poses an interesting challenge. However, a few genetic improvement approaches have tried to allow for decrease of output quality, when improving for other properties, such as energy consumption (Bruce et al. 2018), or shader simplification (Sitthi-amorn et al. 2011). This, however, has not been regarded as a code changing operator itself, but as a side-effect of the other software transformations.

SQL queries were modified a substantial number of times in order to improve execution time. Using search-based techniques to transform SQL queries could allow developers to automatically achieve large improvement to execution time in applications that have many database interactions. Das et al. (2016) found also that not only databases but also file systems led to *speed bugs*, suggesting that I/O could be a good target for improvement.

Many search-based techniques only focus on making changes within a single file. Table 10 suggest that a multi-file approach may be more useful for generating patches to improve non-functional properties. The number of chunks also suggests that patches are distributed widely across source code and changes are found in many locations. Thus, for Android applications, changes could be deliberately spread out across multiple files.

## Related Work

There have been a few studies that mined for non-functional property improving commits in Android applications. Moura et al. (2015) performed a study on software repositories mining “energy aware commits”. They mined commits which attempted to improve the energy efficiency of an application and categorised the commits that did. They used a two-word-key-phrases approach, to narrow down the returned results to 2,189 commits, rather than 112,900 commits that were initially returned from single keyword search. The majority of the commits found in this study ($\sim $70%), belong to categories which concern device configuration, e.g. Voltage Scaling. Das et al. (2016) mined Android repositories for “performance related commits”. They found such messages in only 7% Android application repositories considered. Moreover, their categorisation was more top-level than what we propose, classifying commits into, e.g., “Memory”, “File system”, and other. They have also used one set of keywords for all performance-related issues. Their study restricted their searches to the main application modules. The patterns that they extracted mostly concerned what was being fixed, rather than how it was fixed, making direct comparison difficult. Although there are a few similarities, we both find changes to regular expressions to be useful for improving performance, we find that developers move computation to the background, and we find that caching is used to improve performance. In their work, Das et al. (2016) find that developers improve multiple properties 10.4% of the time, whereas we only found that in 6.5% of cases We have also attempted to use as many keywords as possible, this includes all of the relevant keywords from the related work, and keywords which we determined from analysing commit messages, this is detailed in Section 2.

Mazuera-Rozo et al. (2020) performed a similar study, producing a topology of Android performance bugs, and pointed out several other studies that focused on non-functional bugs. We are concerned with non-functional property *optimisation* rather than *repair*, and thus we describe the types of changes which can improve NFPs, whilst they describe the bugs which can be detrimental to them. In their study they uncovered a number of bug types, for which we have found related fixes. These include the leak fix category, the data structure replacement category, the layout optimisation category. Interestingly, we find around 3-4x the amount of commits dealing with caching for execution time improvement than they do. They also do not capture many of the categories for improving memory usage and bandwidth usage that we do, in particular the Remove Redundancy category. This may be due to our usage of a larger keyword set. As we are concerned with a detailed analysis of the improvement strategies found with respect to four specific performance non-functional property improvement criteria, we use a more comprehensive keyword search than the three aforementioned studies. Consequently, we manually analyse more commits. Moreover, we train a classifier to gather additional data, which we make open source. We also categorise and analyse commits in ways which allow the comparison to existing, and derivation of new, mutation operators for automated software engineering approaches for non-functional property improvement, such as genetic improvement (Hort et al. 2021; Petke et al. 2017).

Linares-Vásquez et al. (2015) attempted to uncover ways in which developers address performance bottlenecks in Android, they did this by surveying developers. They found that developers use similar techniques to the caching and SQL query categories uncovered in this study. Our approach aims to find a more complete picture of the changes developers make by directly exploring the content of the changes they made, as developers may exclude techniques used less frequently when answering a survey.

Chen et al. (2019b) perform a similar study to Mazuera-Rozo et al. (2020), but on 100 projects with C/C++ code. Changes to C/C++ are likely to be different to Java as there is more direct access to lower level operations like memory allocation. However, their arguments pattern is similar to the change parameter pattern that we found and the memorization is similar to our caching pattern. Jin et al. (2012b) conducted a study across a number of different programs in different languages, but did not include Android applications. However they did find fix patterns similar to change parameter, change order of operations, and add condition, showing that some changes made in Android may applicable in other type of software. We also find that changes in Android are larger than those in C/C++. Only a median of lines of code are changed in C/C++ commits, but for all categories we find median changes of 10+ lines and in some cases up to 70+ lines.

Our work uncovers a number of new patterns with respect to those discussed in related work. We find changes to animations, layouts and assets which have not been discussed in previous work, these categories all seem to be particularly relevant UI focused Android applications. We also find that searching for source code changes results in a number of patterns that have not been discussed in previous work, like remove redundancy, early return, different library, data structure changes and time out reduction.

The idea of mining repositories for software improving commits has shown promise in the automated program repair field. Long et al. (2017) mined Java repositories to automatically find fix patterns to apply to buggy software. Kim et al. (2013) manually evaluated human written patches from GitHub to generate patches which could then be automatically applied to buggy software. Bader et al. (2019) leverage the version history of a piece of software to extract fix patterns to suggest to developers. Martinez and Monperrus (2018) showed that repair templates generated by mining existing code could be used to generate large number of bug fixes. Several of the automated program repair techniques mentioned use of search-based approaches, which have been utilised for improvement of non-functional properties, in the field of genetic improvement (Petke et al. 2018), in particular. Therefore, we believe that the results of our study could be similarly used to provide recommendations for future tooling (Petke 2017).

## Threats to Validity

Although we cannot claim that our results can apply to any type of mobile application, we mitigate the conclusion validity threat by mining commits from applications diverse in many ways, including their type, size, number of commits made and number of contributors.

Moreover, developers of open-source applications may view non-functional properties at a different priority than their closed source counterparts. However, the user base is the same (Android mobile users), and open-source repositories provide a rich amount of data that can be analysed to improve current software engineering practice. The number of NFP improving commits we analysed may give a partial picture of the commits which are actually made by developers, and it was limited by the manual effort needed to analyse the 3,132 commits. However, this is in par with the number of commits examined in previous studies (e.g., Moura et al.^2015^; Das et al. 2016). Some commits which improve NFPs may have been missed by the keyword search. This may have been due the sets of keywords used being incomplete or the commits not having messages which reflect the non-functional properties which they improve. To mitigate the former, the keywords were repeatedly expanded to try to catch as many commits as possible and had a very large false positive rate (73%), so it is unlikely that many were missed. It is also likely that developers did not report trade-offs that were made. The classifier may also be influenced by the keywords used, as it was trained on the gold set based on keywords search and manual analysis. We do provide the source code of both the keyword search and classifier (github.com/SOLAR-group/NonFunctionalAndroidCommits), so that they can be expanded in the future.

Finally, the NFP improving commits could be categorised differently. The aim of the current categorisation was to provide meaningful classes from which insights could be drawn towards possible new mutation operators for non-functional property improvement using search-based approaches, such as genetic improvement (Petke et al. 2018). To alleviate such a threat and facilitate this aim, we provide our detailed commit categorisation on-line (github.com/SOLAR-group/NonFunctionalAndroidCommits).

## Conclusions and Future Work

In this paper we have explored the ways in which Android application developers improve non-functional properties, in order to guide the development of new automatic transformations to improve the NFPs of Android applications. To this end we analysed 74,408 commits from 100 repositories, finding 560 commits that improve four performance-related non-functional properties (NFPs): *execution time*, *memory consumption*, *bandwidth usage*, and *frame rate*. We employed a combination of keyword search, and a classifier to filter irrelevant commits, and manually analysed over 3,000 commits to obtain our corpus. We have found that developers more commonly improve the execution time of their applications than other NFPs. However, manual changes to improve non-functional properties are uncommon, suggesting that automated tools could aid developers in this challenging task. Moreover, we find that developers occasionally improve multiple non-functional properties at once (5.2% of cases), or sacrifice one property for another (10.7% of cases). This suggests developers are willing to take multi-objective approaches. However, the rarity of these changes suggests a need for automatic tools that could propose a Pareto of solutions for the developer to choose from (Harman et al. 2010). We have also found similarity between 5 current mutation operators in automated software improvement and the changes that app developers make. Code removal was a very common technique used by app developers for improving multiple non-functional properties, as was data structure replacement. It would be interesting to explore whether the corresponding mutation operators, which have already been successfully used to optimise NFPs of traditional software, are effective in automatically improving NFPs of mobile applications too. We have also found novel ways in which real commits could be more closely mimicked by tools for automated NFP improvement, for example, by making changes across multiple files simultaneously.

Our results highlight a need for automated tools which improve the non-functional properties of Android applications, and provide initial guidance on what types of changes such tools could implement. These changes include automatic caching to improve speed and bandwidth, SQL query transformation to improve speed, and image modification to reduce memory consumption and execution time.

Our study could be further extended. First of all, our corpus could be extended in terms of commit types and size. Commits improving other non-functional properties such as code quality could be collected and analysed in future studies. Future work could use automatic pattern recognition from a large set of NFP improving commits, to generate generic patterns improving NFPs of Android applications.

## Data Availability

All code, including the mining scripts and the classifier, is also available at github.com/SOLAR-group/NonFunctionalAndroidCommits.

## Appendix A: Classifier Training

In order to build an accurate classifier for NFP improving commits, we investigated several different options as detailed in this Appendix. The source code and the results obtained are provided in our on-line repository (github.com/SOLAR-group/NonFunctionalAndroidCommits). For clarity we compare between three different classes. *unknown* are commits that have not been analysed, and were excluded by the keyword search. *irrelevant* commits were identified via keyword search, but deemed not relevant towards non-functional properties by one or more of the examiners. Finally, *relevant* commits encompass all commits identified to deal with a non-functional property after keyword search.

We initially attempted to generate the classifier for all four subclasses of relevant commits, execution time, memory, bandwidth and frame rate. This yielded no satisfactory results as the classes bandwidth and framerate had no recall (not a single commit) and the other two groups yielded less than 0.1 recall (i.e., less than 1 in 10 commits found).

We also attempted balancing the data. This was done in two ways. The first was to balance the *irrelevant* commits (3,132) with the *relevant* class (229) as the drastic difference lets classifiers overfit towards the *irrelevant* class. The balancing yields large margins in the test and training sets (accuracy 0.8, with the same recall in both classes). However, when attempting to reproduce this on the entire dataset the precision in the relevant classes dropped to 0.07. In this case the classifier misses 20% of commits, but yields no advantage over keyword search as many commits that are not relevant are identified as such. The second attempt was balancing the classes of commits to reduce the under-representation of bandwidth and frame rate (see Table 6). This reduced the recall to 0.02 meaning that the classifier finds only 2 in 100 commits. We also investigated algorithms specifically targeted towards unbalanced data, also to no avail. All of the balancing was done by keeping all commits of the respective smallest class, and randomly removing commits from other classes until they were the same size.

Since balancing the data worsens the precision drastically, we continued training classifiers with the datasets as they were recorded (i.e., without balancing). This investigation was conducted with all combinations of text preprocessing and featurisation. In all cases we did a preliminary tokenisation and stop word removal of all commit messages. For string processing we used:

- Lemmatisation—reducing word inflections to a word root
- Stemming—removing word endings to approximate word roots

For the featurisation of the remaining word roots we used:

- TF/IDF—term frequency / inverse document frequency essentially ranking words in the source
- Bag of words—counting words in the commit messages
- Improved bag of words—the adaption was that we reduce to words that we identified as discriminative between the *relevant* and *irrelevant* sets. E.g. when a word occurs more often in one or the other it is included, otherwise it is not used in bag of words.

Table 15 shows the best classifier found with this. Quadratic Discriminant Analysis yields a recall of 0.68 meaning two in three commits are identified, and a precision of 0.36 meaning that about three commits have to be consulted manually to yield one relevant commit. This is much better than keyword search (13,67 commits on average) but still represents a loss of information. The best results were yielded via using Lemmatisation as preprocessing step and improved bag of words to create the feature vectors.

**Table 15:** Quadratic discriminant analysis of NFP finding two in three commits

	Precision	Recall	F1-score
Relevant	0.36	0.68	0.47
Irrelevant	0.97	0.90	0.94

Quadratic Discriminant Analysis was the best algorithm found out of the following twenty algorithms:

- Multi-layer Perceptron Classifier
- C-Support Vector Classification
- Linear C-Support Vector Classification
- Gaussian Process Classifier
- Decision Tree classifier
- Extra Tree Classifier
- Random Forest Classifier
- Ada Boost Classifier
- Bagging Classifier
- Gaussian Native Bayes
- Multinomial Native Bayes
- Bernoulli Native Bayes
- Complement Native Bayes
- Quadratic Discriminant Analysis
- Linear Discriminant Analysis
- Stochastic Gradient Descent Classifier
- Ridge Classifier
- Passive Aggressive Classifier

We chose Quadratic Discriminant Analysis as it supports the highest recall in the relevant category while still being a reasonable filter. Multinomial Native Bayes has the highest accuracy overall (0.95) but only a recall of 0.32, meaning two out of three *relevant* commits are lost. Quadratic Discriminiant Analysis is highly dependent on the feature selection, as selecting regular bag of words actually produces a recall of 1.0 for relevant commits (no loss), but with a precision of 0.09, meaning that it requires an average of 11 commits to find one relevant one. This is similar to the results that the keyword search itself produces. Further information is captured in the GitHub repository.

In an attempt to improve the quality of our classifier we then added the dataset from Mazuera-Rozo et al. (2020), which are the results discussed in the main body of this work. The results of this analysis are available in our online repository consisting of csv tables with 10 runs of a random training / test split and a final line with the averages over all runs. All of the Classifiers using TF/IDF are outperformed by classifiers using other featurisation methods. In a similar vein lemmatisation outperformed stemming. In general our improved bag of words slightly outperforms bag of words. The exception is that best classifier Decision Tree which performs best when using TF/IDF with stemming. It achieves a recall of 0.80 (i.e. 4 out of 5 commits) with a precision of 0.72 meaning that about 3 in 4 commits will be found.

## Appendix B: Algorithm Changes in Commits

In this Appendix we describe the commits in the “other” subcategory in the “Different algorithm” category, found in Section 3.2, in more detail. We First describe those from the KM set, then those from the CM set. For each commit, we report its SHA and a description of the changes made in it.

### B.1 KM Commits

- **c9afd823e8da9393a167a89345301782ef3483b**: Change from depth first search to depth last.
- **f3974898af9299632ea9354accb61e12393308c2**: Use a look up table.
- **b0e4f59f43984867c123fbffb4f345d5cc5f3814**: Change if else statement to a switch statement.
- **85ead3bd940bd445bbaff6a4a4d30ebca6b7c7a6**: Use the spread operator.
- **22904667b8e79167339972d4682024d95cd3d169**: Use lazy initialisation.

### B.2 CM Commits

- **fc82441c9aa412be6c1448b99a34607ff98e551d**: Use the SharedPreferencesCompat apply method.
- **588c35967f3dd9c2d27bb8739c46922a9b7a1c24**: Call free methods of class components in their free methods, rather than in the onClose method of the class.
- **1cc32362aad23dfe5d508776274e643a307a3577**: Use lzma2 compression.
- **8bc2b8d5f53b04bbaea49a52eab260e02684376e**: Use String.format.
- **adbcdddb5625d7cd49b80d5c560eb8998446183a**: Faster text rendering method, blitFrom instead of addBlitFrom.
- **1aedaa5c28fc6a3cae76c34ac03d808d5860aa1c**: Faster algorithm for calculating the position of elements on screen.
- **a9bc9ed71a84d68bf3e1652550251320c0a38cd8**: Use getCount, rather than iterating over whole cursor.
- **d9c393f20e4d869628d8e3531af4e63a4e7b851b**: Use Androidx workManager begin to en-queue jobs.
- **5731360f8a894e49c5383857b629e450af9fa29d**: change DOM to SAX for UML parsing.
- **893909146d6e406ca36c5f2d95b82526a4ed6167**: Use raw input (effects rooted devices only).
- **6d1fa0cf056289457c3ca7616861a3dd362caea7**: More efficient average calculation.

## Footnotes

1 In comparison, Moura et al. (2015) found 371 energy-aware commits from a sample of 2,189 curated commits. It should be noted these span different numbers of repositories, and different keywords, corresponding to relevant non-functional software properties.

2 We omit energy commits, as very similar studies targetting these have already been conducted (e.g., Moura et al. 2015), with Banerjee and Roychoudhury (2016) already implementing a refactoring tool for energy bugs.

3 https://fossdroid.com/

4 The keywords taken from previous work were: ‘wait’, ‘tim’, ‘stuck’, ‘react’, ‘latenc’, ‘throughput’, ‘suboptimal’, ‘bloat’, ‘utilization’, ‘ANR’, ‘OOM’, ‘bottleneck’, ‘hot-spot’, ‘length’, ‘consumption’

5 We decided to group all relevant commits into one single group, as preliminary analysis showed that attempting to classify the commits into multiple classes (i.e., execution time, memory, bandwidth and frame rate) produces classes that are too small for building an accurate classification model (the Recall in all groups was less than 0.1).

6 We considered also using issue messages to identity commits. However the analysis of our KM data set showed that only 13% of commits had associated issues. Most (52%) of those issues were associated with 10 or more commits, meaning that only a small fraction of their messages and comments would be related to the commit that we are interested in.

7 We had to set a limit on the number of repositories due the manual effort required to analyse the precision of the classifier.

8 We note that a lot of these were small repositories, as the 40 repositories in which NFP improving commits were found had altogether 39,420 commits, while the other 39 had altogether 6,958 commits.

9 An example of a commit which could not be classified can be found at https://github.com/ar-/apple-flinger/commit/bbc70cdc0a8190153195f46fe8c873def6ca3e98

10 https://developer.android.com/studio/write/lint

11 https://fbinfer.com/

12 https://github.com/Yelp/android-varanus
